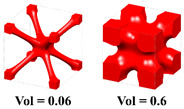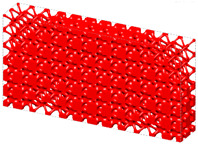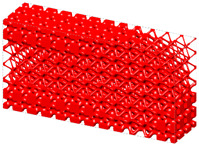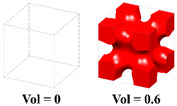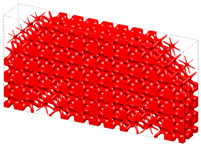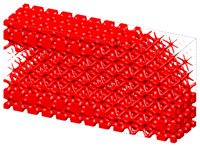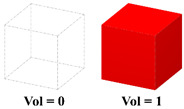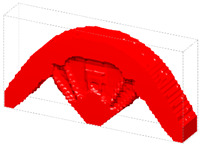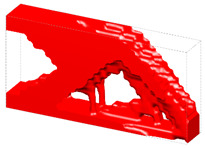# A Hybrid Level Set Method for the Topology Optimization of Functionally Graded Structures

**DOI:** 10.3390/ma15134483

**Published:** 2022-06-25

**Authors:** Junjian Fu, Zhengtao Shu, Liang Gao, Xiangman Zhou

**Affiliations:** 1 Hubei Key Laboratory of Hydroelectric Machinery Design & Maintenance, Yichang 443002, China; fjj@ctgu.edu.cn (J.F.); zhouxman@ctgu.edu.cn (X.Z.); 2 College of Mechanical and Power Engineering, China Three Gorges University, Yichang 443002, China; 3 State Key Laboratory of Digital Manufacturing Equipment and Technology, Huazhong University of Science and Technology, Wuhan 430074, China; gaoliang@mail.hust.edu.cn

**Keywords:** topology optimization, hybrid level set method, thin-walled stiffened structures, functionally graded cellular structures

## Abstract

This paper presents a hybrid level set method (HLSM) to design novelty functionally graded structures (FGSs) with complex macroscopic graded patterns. The hybrid level set function (HLSF) is constructed to parametrically model the macro unit cells by introducing the affine concept of convex optimization theory. The global weight coefficients on macro unit cell nodes and the local weight coefficients within the macro unit cell are defined as master and slave design variables, respectively. The local design variables are interpolated by the global design variables to guarantee the C^0^ continuity of neighboring unit cells. A HLSM-based topology optimization model for the FGSs is established to maximize structural stiffness. The optimization model is solved by the optimality criteria (OC) algorithm. Two typical FGSs design problems are investigated, including thin-walled stiffened structures (TWSSs) and functionally graded cellular structures (FGCSs). In addition, additively manufactured FGCSs with different core layers are tested for bending performance. Numerical examples show that the HLSM is effective for designing FGSs like TWSSs and FGCSs. The bending tests prove that FGSs designed using HLSM are have a high performance.

## 1. Introduction

Functionally graded structures (FGSs) [1] are novel structures with spatially varying characteristics. Their microstructures and mechanical properties exhibit graded changes in their preferred orientation. Graded design can provide an excellent mechanical performance, including a high specific stiffness, good anti-bend performance, and good energy absorption properties [2,3]. Therefore, FGSs are excellent design carriers for realizing various functions, including load-bearing, vibration isolation, and heat protection [4]. A large number of man-made FGSs, including thin-walled stiffened structures (TWSSs) with graded ribs and functionally graded cellular structures (FGCSs), have been widely used in aerospace [5,6], ships [7], and medical implants [8]. It is worth noting that the mechanical properties of such lightweight structures mainly depend on their structural geometry configurations [9]. Topology optimization [10] is an intelligent design method that can provide great design freedom to obtain unexpectedly high-quality and innovative configurations. Several topology optimization methods have been proposed to achieve the optimal design of TWSSs and FGCSs, respectively. However, existing methods are more pertinent. Fewer related studies can simultaneously realize the topology optimization of the aforementioned FGSs. Additionally, scale effects, geometric continuity, and manufacturing constraints need to be resolved in the topology optimization so that the optimized results are more practical for engineering purposes. Therefore, a general and effective topology optimization method is still required to optimize 3D FGSs for better performance and functions.

TWSSs are an integral component fabricated by connecting panels and stiffening ribs. By properly designing the layout of stiffening ribs on the surface of a thin plate, the strength, stiffness, impact and fatigue resistance of the TWSSs [11] can be significantly improved, while ensuring that they are lightweight. Generally, it is difficult to reduce material consumption and improve the mechanical properties of TWSSs with a uniform stiffening ribs layout. This design strategy is an effective way to solve the above problems by setting graded stiffening ribs with a variable stiffness. At present, most research focuses on transforming the layout design problem of stiffening ribs to find the optimal distribution of materials, including the variable thickness method [12,13], base structure method [14,15], adaptive growth method [16,17], etc. The essential idea of determining the optimal distribution of materials is similar to that of topology optimization. To further satisfy the design requirements and comprehensively improve mechanical properties, concurrent topology optimization methods are proposed for the design of hierarchical TWSSs [18,19]. The size, shape and position of the stiffening ribs are determined but with less consideration for manufacturing constraints. Hence, how to realize the optimal design of TWSSs with favorable mechanical properties and an excellent manufacturability has always been one of the most challenging topics in this field.

The FGCS contains a quantity of graded microstructure unit cells, which exhibit better mechanical properties than periodically repeated cellular structures [20,21]. Lattice sandwich structure (LSSs) are a special class of cellular structures that are fabricated by attaching two thin but stiff layers to lightweight but thick cores [22]. Topology optimization methods based on solid isotropic materials with penalization (SIMP) [23,24,25], the level set method (LSM) [9,26,27], and bidirectional evolutionary structural optimization (BESO) [28,29,30] have been successively proposed for designing FGCSs. Specifically, the multi-scale topology optimization method [31] is the mainstream design method for FGCSs, which can comprehensively optimize the distribution of macrostructure materials and their spatially varying microstructure configurations. However, due to the introduction of the homogenization method [32] to evaluate the equivalent properties of microstructures, the scale separation effects and non-continuity of microstructures are difficult to resolve. To deal with these challenges, the scale-related topology optimization methods [20,33,34] of FGCSs are proposed by building a unified finite element model between the macro and micro-topology structure. Compared with scale-separated topology optimization, design methods with scale-related features have more engineering applicability.

FGSs have various forms and complex geometric features. The common design characteristic of different FGSs is that the macrostructure contains numerous microstructure unit cells. Hence, this paper constructs a design strategy to drive the material distribution of macrostructures through the topology optimization of microstructures. Additionally, a hybrid level set method (HLSM) is proposed for the topology optimization of FGSs. By presetting the level set functions of various structural unit cells, high-performance FGSs with different structural forms and functional properties are obtained. This also shows that HLSM has significant advantages for the topology optimization of various FGSs. Numerical examples of TWSSs and FGCSs are given. Meanwhile, FEA and mechanical experiments verify the feasibility and effectiveness of the proposed method.

## 2. Geometric Modeling Based on HLSM

### 2.1. Level Set Modeling

The LSM provides a flexible and convenient implicit modeling method for structural representation with complicated geometric characteristics. The structure represented by the level set function (LSF) has creep geometric boundary, which is convenient for further manufacturing process. The LSF is thus applied to describe the geometric model of the FGSs. The basic idea of the LSM is to describe the structural boundary with a higher-dimensional implicitly function *Φ*(***X***). The dynamic structural interfaces are obtained by tracking the movement of the higher dimensional function. Its mathematical formulation is defined as follows [35,36]:

(1) ∂Ω(X)={X:Φ(X)=0,X∈D}

where ***X*** is the physical spatial coordinate vector of the higher-dimensional space. D is the design domain that contains the complete structure. ∂Ω = **Γ***_D_*∪**Γ***_N_*∪**Γ***_f_* contains Γ*_D_* as the Dirichlet boundary, Γ*_N_* as the Neumann boundary and Γ*_f_* as the traction free boundary.

The solid part, interface and empty part are described by the following definition:

(2) $$\left\{ \begin{array}{l} \Phi (X)>0,\;\;X\in \Omega \\ \Phi (X)=0,\;\;X\in \partial \Omega \\ \Phi (X)<0,\;\;X\in D\backslash \Omega \end{array}\right.$$

where Ω is the structure domain. ∂Ω is the interface of the structure implicitly represented as the zero contour of LSF *Φ*(***X***).

**Figure 1:**
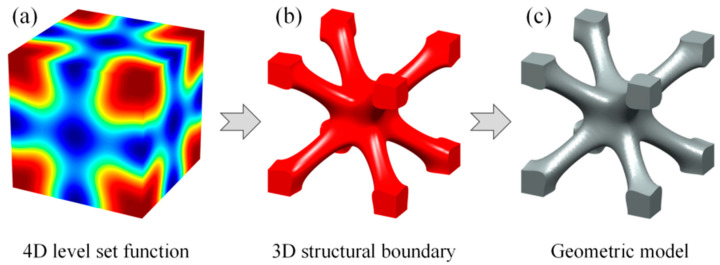
Implicit modeling based on LSF and 3D geometric model. (**a**) The LSF. (**b**) Contour of zero level set. (**c**) 3D geometric model.

This paper introduces the triply periodic minimal surface (TPMS) [37] cell as one of the basic unit cells. The TPMS is mathematically and implicitly modeled by a 4D LSF. The lower-dimensional contour is actually the 3D structural boundary of a specific cellular structure. Figure 1 illustrates the implicit level set representation of the I-Wrapped Package (IWP) TPMS [38]. A parametric cellular structure with varying geometrical shape and volume fractions can be obtained by taking different contours.

**Figure 2:**
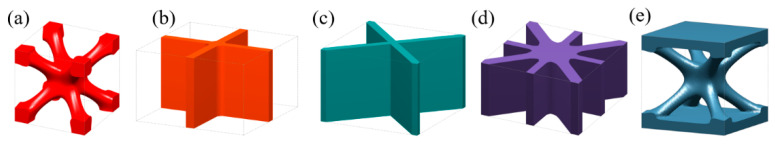
Unit cells constructed by LSF. (**a**) Triply periodic minimal surface (I-WP). (**b**) Cross-shaped stiffening rib unit cell. (**c**) X-shaped stiffening rib unit cell. (**d**) Hybrid stiffening rib unit cell. (**e**) Lattice sandwich structure unit cell.

The symbolic distance function (SDF) [39] can be used as the LSF *Φ*_p_(***X***) of the geometric structures. The SDF is given as:

(3) $$\Phi_{P}(X)=\left\{ \begin{array}{l} +\underset{X_{b}\in \partial \Omega P}{\min}\;\left\|X-X_{b}\right\|\;\forall X\in \Omega_{P}\\ -\underset{X_{b}\in \partial \Omega P}{\min}\;\left\|X-X_{b}\right\|\;\forall X\in D\backslash \Omega_{P} \end{array}\right.$$

where Ω describes the structure domain. ∂Ω is the interface implicitly represented by the zero contour of LSF *Φ*(***X***). ***X***_b_ is any point on the curve (or surface). ||•|| is the Euclidean norm. In addition, explicit modeling techniques have certain limitations in the parametric modeling of the microstructure with complex geometric features and rich variations. Theoretically, any type of microstructures can be digitally constructed based on the SDF. For example, using the above implicit modeling strategy, some microstructure unit cells with different topological forms are built for various FGSs, as shown in Figure 2.

### 2.2. Hybrid Level Set Function

**Figure 3:**
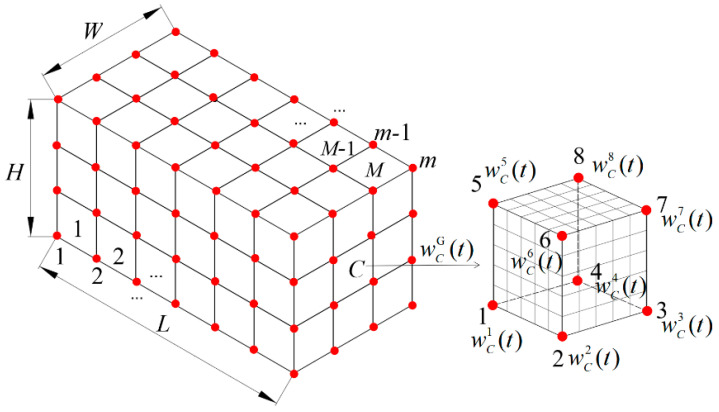
Definition of design variables.

Different from the traditional single-scale LSM, the design domain needs to be discretized into multiple unit cells in the proposed HLSM. Then, pre-defined unit cells with varying forms should match them. There are similar processing operations in previous studies of multi-scale topology optimization methods [40]. In the global hierarchy, the design domain is discretized by *M* eight-node unit cells. It is assumed that there are *m* nodes on the discrete design domain. The global weight coefficients *w*(*t*) are the master design variables defined on the unit cell nodes, as illustrated in Figure 3. On the local hierarchy, each unit cell, for example *C*, is further discretized by eight-node hexahedral elements with a number of nodes. Slave design variables, i.e., local weight coefficients, are defined on element nodes within the unit cell.

The global weight coefficient ***w***(*t*) is an *m* × 1 time-dependent vector:

(4) $$w(t)={\left[w_{1}(t)\;w_{2}(t)\;w_{3}(t)\;\cdots \;w_{m}(t)\right]}^{Τ}$$

The weight coefficient $w_{C}^{G}$(*t*) of a unit cell is a 8 × 1 (for 3D case) time-dependent vector. Meanwhile, the weight coefficient $w_{C}^{G}$(*t*) is also a sub-vector of the global weight coefficient vector ***w***(*t*):

(5) $$w_{C}^{G}(t)={\left[w_{C}^{1}(t)\;w_{C}^{2}(t)\;w_{C}^{3}(t)\;w_{C}^{4}(t)\;w_{C}^{5}(t)\;w_{C}^{6}(t)\;w_{C}^{7}(t)\;w_{C}^{8}(t)\right]}^{Τ}=S_{C}w(t)$$

where ***S****_C_* is a selection matrix used to select the weight coefficient $w_{C}^{G}$(*t*) of the unit cell from the global weight coefficient ***w***(*t*). The dimension of the selection matrix ***S****_C_* is 8 × *m*, which is defined as follows:

(6) $$S_{C}=\;{\left[\begin{array}{ccccccccccccccccc} \cdots & 1 & \cdots & 0 & \cdots & 0 & \cdots & 0 & \cdots & 0 & \cdots & 0 & \cdots & 0 & \cdots & 0 & \cdots \\ \cdots & 0 & \cdots & 1 & \cdots & 0 & \cdots & 0 & \cdots & 0 & \cdots & 0 & \cdots & 0 & \cdots & 0 & \cdots \\ \cdots & 0 & \cdots & 0 & \cdots & 1 & \cdots & 0 & \cdots & 0 & \cdots & 0 & \cdots & 0 & \cdots & 0 & \cdots \\ \cdots & 0 & \cdots & 0 & \cdots & 0 & \cdots & 1 & \cdots & 0 & \cdots & 0 & \cdots & 0 & \cdots & 0 & \cdots \\ \cdots & 0 & \cdots & 0 & \cdots & 0 & \cdots & 0 & \cdots & 1 & \cdots & 0 & \cdots & 0 & \cdots & 0 & \cdots \\ \cdots & 0 & \cdots & 0 & \cdots & 0 & \cdots & 0 & \cdots & 0 & \cdots & 1 & \cdots & 0 & \cdots & 0 & \cdots \\ \cdots & 0 & \cdots & 0 & \cdots & 0 & \cdots & 0 & \cdots & 0 & \cdots & 0 & \cdots & 1 & \cdots & 0 & \cdots \\ \cdots & 0 & \cdots & 0 & \cdots & 0 & \cdots & 0 & \cdots & 0 & \cdots & 0 & \cdots & 0 & \cdots & 1 & \cdots \end{array}\right]}_{8\times m}$$

where the values in the omitted positions are 0.

Weight coefficients within the unit cell are defined by the interpolation of $w_{C}^{G}$(*t*) using the common shape functions. For convenience, the shape function vector ***N***_C_(***x***) is written as:

(7) $$N_{C}(x)=[N_{C}^{1}(x)\;N_{C}^{2}(x)\;N_{C}^{3}(x)\;N_{C}^{4}(x)\;N_{C}^{5}(x)\;N_{C}^{6}(x)\;N_{C}^{7}(x)\;N_{C}^{8}(x)]$$

For an eight-node hexahedral element, the shape function $N_{C}^{\;i}$ is defined as:

(8) $$N_{C}^{\;i}(x)=\frac{1}{8}(1+\xi_{0})(1+\eta_{0})(1+\zeta_{0})\;i=1,2,3,\cdots ,8$$

where *i* is the number of unit cell nodes. *ξ*_0_ = *ξ_i_ξ*, *η*_0_ = *η_i_η*, *ζ*_0_ = *ζ_i_ζ*, *ξ_i _*= ±1, *η_i_* = ±1, and *ζ_i_* = ±1. *ξ*, *η* and *ζ* are the internal node coordinates of the unit cell in local coordinate system.

According to Equations (6)–(8), the global weight coefficient vector ***w***_C_(*t*) of the unit cell is given as:

(9) $$w_{C}(t)=N_{C}w_{C}^{G}(t)=N_{C}S_{C}w(t)$$

In order to realize the geometrically graded evolvement of FGSs with different shapes and enrich the layout configurations, this paper introduces the concept of affine in convex optimization theory [41] to construct the HLSF. The affine concept is defined as: a set *Q* ⊆ *R* is an affine if there is a line through any two distinct points in *Q*, i.e., if for any *x*_1_, *x*_2_ ∈ Q and *s*∈R, we have *sx*_1_ + (1 − *s*)*x*_2_ ∈ Q. In a similar concept, the HLSF of two pre-defined unit cells can generate a new unit cell with a different geometrical configuration and volume fraction. The hybrid process of unit cells is defined as follows:

(10) $$\Phi_{H}(X)=s\Phi_{1}(X)+(1-s)\Phi_{2}(X)$$

where *Φ*_H_(***X***) is the LSF of the unit cell with new structural features. *Φ*_1_(***X***) and *Φ*_2_(***X***) are the LSFs of two pre-defined master unit cells, respectively. ***s*** is the design variable, which is the weight coefficient vector of the two pre-defined unit cells.

Based on Equation (10), the HLSF is constructed by introducing a weight coefficient to realize the parametric modeling of FGSs. The HLSF is defined as follows:

(11) $$\left\{ \begin{array}{l} \Phi (x,t)=w(t)\varphi_{1}(x)+(1-w(t))\varphi_{2}(x)>0,\;\forall x\in \Omega /\partial \Omega \\ \Phi (x,t)=w(t)\varphi_{1}(x)+(1-w(t))\varphi_{2}(x)=0,\;\forall x\in \partial \Omega \\ \Phi (x,t)=w(t)\varphi_{1}(x)+(1-w(t))\varphi_{2}(x)<0,\;\forall x\in D/\Omega \end{array}\right.$$

where the weight coefficient ***w***(*t*) is a variable vector with respect to the pseudo-time *t*, and the value range is 0 ≤ ***w***(*t*) ≤ 1. *φ*_1_(***x***) and *φ*_2_(***x***) are the LSFs of two pre-defined unit cells, respectively. The pseudo-time *t* adds dynamics to the weight coefficient ***w***(*t*), indirectly changing the implicit interface *∂*Ω.

**Figure 4:**
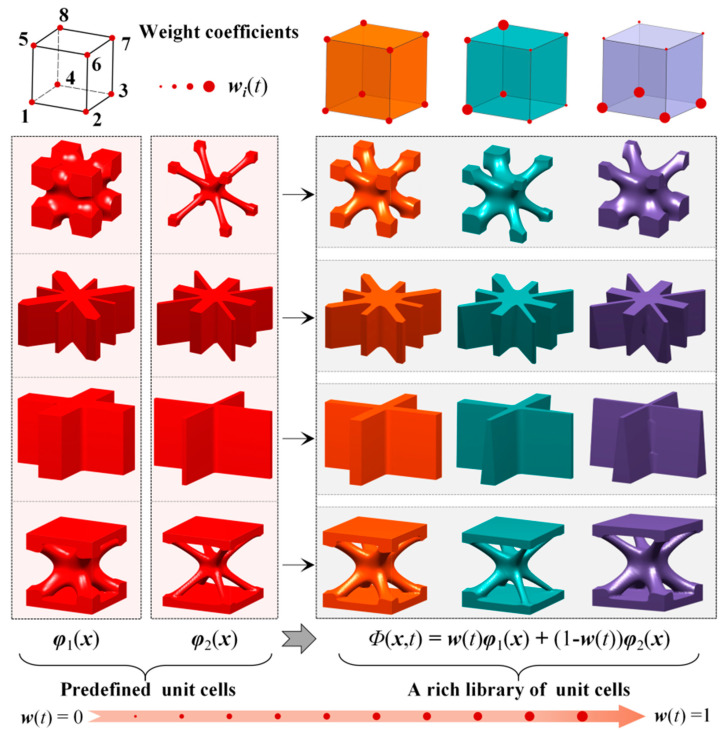
Illustration of FGSs unit cells generated with the HLSF.

The hybrid process of the LFSs is actually a linear weight interpolation, as illustrated in Figure 4. The desired graded mechanical performance of the unit cell is obtained by optimizing the corresponding weight coefficients. Additionally, the influence of the weight coefficients on a microstructure configuration is divided into two cases: (1) When the values of the weight coefficient matrix ***w***(*t*) are all the same, the geometric configuration of the new unit cell changes uniformly in space. (2) Conversely, when the values of the ***w***(*t*) are different, the geometry of the unit cell takes on an asymmetric evolution. Therefore, when the weight coefficients correspondingly change with the external loading and boundary conditions during the optimization process, a series of microstructure unit cells with diverse geometries are distributed in the macrostructure. The obtained unit cells can exhibit directional stiffness, and thus provide enormous design freedom for the optimal design of the FGSs.

**Figure 5:**
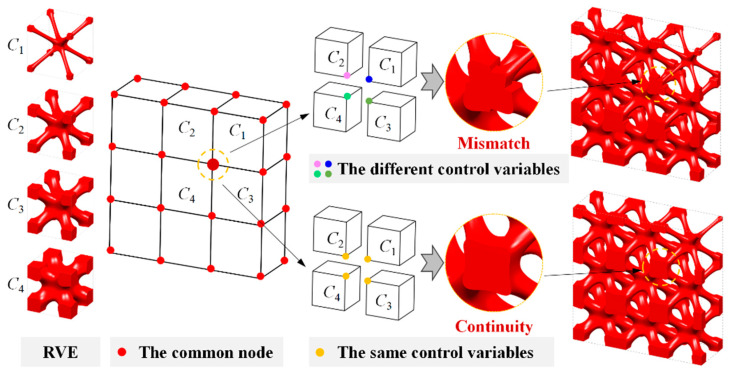
Illustration of the continuity of geometric boundaries.

Based on the definition of the weight coefficient vector ***w****_C_*(*t*), the continuous geometry transition of neighboring unit cells can be expected. For example, I-WP TPMSs are taken as the representative volume elements (RVEs) to illustrate the interpolation process, as shown in Figure 5. *C*_1_–*C*_4_ are RVEs with different volume fraction. This diagram also demonstrates the basic idea of the strategy of geometrical continuity. The geometry continuity of neighboring unit cells depends on the continuity of the weight coefficient vector ***w****_C_*(*t*). During the optimization process, the local design variables are updated by the interpolation of global design variables. According to the definition in Figure 3, neighboring unit cells share the same weight coefficients on common boundaries. Thus, the interpolation function the global weight coefficients have at least C^0^ continuity. With this updating strategy of design variables, the proposed HLSM can naturally guarantee geometry continuity without imposing any extra constraints.

## 3. Topology Optimization Model and Sensitivity Analysis

### 3.1. Topology Optimization Model

For simplicity, this paper only considers the static stiffness maximization problem. The mathematical formulation of the topology optimization model based on the HLSM is given as:

(12) $$\begin{array}{c} \mathrm{find}:\;{w(t)=(w_{1}(t)\;w_{2}(t)\;w_{3}(t)\;\cdots \;w_{m}(t))}^{T}\\ \min:\;J(\Phi )=\frac{1}{2}\int_{\Omega }\varepsilon^{T}(u)E\varepsilon (u)d\Omega \\ s.t\{\begin{array}{c} a_{\Phi }(u,v)=l_{\Phi }(v),\;\;\forall v\in U\\ g(\Phi )=\int_{\Omega }d\Omega -\mu \overset{-}{V}\leq 0\\ 0\leq w_{k}(t)\leq 1 \end{array} \end{array}$$

where *J*(*Φ*) is the objective function. ***ε*** is the strain field. ***u*** is the displacement field. ***E*** is the material elasticity tensor of solid material. *a_Φ_*(***u***,***v***) and *l_Φ_*(***v***) are the bilinear and linear term of the state equation of the linear system.***v*** is the virtual displacement field belonging to the space U spanned by the kinematically admissible set of displacement. *μ* is the prescribed volume fraction. *g*(*Φ*) is volume of the whole design domain. *w_k_*(*t*) represents the global weight coefficient of the *k*’th node in the design domain, *k* is the number of discrete element nodes in the design domain.

The equilibrium equation *a_Φ_*(***u***,***v***) = *l_Φ_*(***v***) is given in its weak form. The energy bilinear term and load liner term are defined as:

(13) $$a_{\Phi }(u,v)=\int_{\Omega }\varepsilon^{Τ}(u)E\varepsilon (v)d\Omega$$

(14) $$l_{\Phi }(v)=\int_{\Omega }fvd\Omega +\int_{D}pv\delta (\Phi )\left|\nabla \Phi \right|d\Omega$$

where *f* is the traction force on the boundary and *p* is the body force density. |ᐁ*Φ*| is the norm of gradient of LSF. *δ*(*Φ*) is the derivative of the Heaviside function, known as the Dirac function, which is given as:

(15) $$\delta \left(\Phi \right)=\{\begin{array}{c} \frac{3(1-\gamma )}{4\Delta }\left(1-\frac{\Phi^{2}}{\Delta^{2}}\right)\;\left|\Phi \right|\leq \Delta \\ 0\;\;\;\;\;\;\;\;\;\;\;\;\;\;\;\;\;\;\;\;\;\;\;\;\;\;\;\left|\Phi \right|>\Delta \end{array}$$

where *γ* = 0.001 is a small positive number. Δ is half of the bandwidth.

### 3.2. Sensitivity Analysis

Under the theoretical framework of the LSM, the shape derivative theory [42] is used to derive the velocity field of the boundary motion of the level set. A similar method can also be applied to the HLSM to derive the sensitivity of the objective function and constraint conditions with respect to design variables.

The shape derivative of the objective function, the energy bilinear form, and the load linear form with respect to the pseudo-time variable *t* are calculated by:

(16) $$\frac{\partial J(\Phi )}{\partial t}\;=\int_{\Omega }\varepsilon^{Τ}(\dot{u})E\varepsilon (u)d\Omega +\frac{1}{2}\int_{\Omega }\varepsilon^{Τ}(u)E\varepsilon (u)\delta (\Phi )\frac{\partial \Phi }{\partial t}d\Omega$$

(17) $$\frac{\partial_{\Phi }a(u,v)}{\partial t}=\int_{\Omega }\varepsilon^{Τ}(\dot{u})E\varepsilon (v)d\Omega +\int_{\Omega }\varepsilon^{Τ}(u)E\varepsilon (\dot{v})d\Omega +\int_{\Omega }\varepsilon^{Τ}(u)E\varepsilon (v)\delta (\Phi )\frac{\partial \Phi }{\partial t}d\Omega \;$$

(18) $$\frac{\partial_{\Phi }l(v)}{\partial t}=\int_{\Omega }[f\;\dot{v}+div(p\dot{v}n)]d\Omega +[f\;v+div(pvn)]\delta (\Phi )\frac{\partial \Phi }{\partial t}d\Omega$$

where ***n*** is the normal vector.

Since $\dot{v}\;$∈ *U*, the conjugate equation can be obtained:

(19) $$\int_{\Omega }\varepsilon^{Τ}(u)E\varepsilon (\dot{v})d\Omega \;=\int_{\Omega }\left[f\;\dot{v}+div(p\dot{v}n)\right]d\Omega \;$$

The partial derivatives of both sides of the equilibrium equation can be found with regard to pseudo-time *t:*

(20) $$\frac{\partial a_{\Phi }(u,v)}{\partial t}=\frac{\partial l_{\Phi }(v)}{\partial t}$$

By substituting Equations (17)–(19) into Equation (20):

(21) $$\int_{\Omega }\varepsilon^{Τ}(\dot{u})E\varepsilon (v)d\Omega =\int_{\Omega }[fv+div(pvn)d\Omega -\varepsilon^{Τ}(u)E\varepsilon (v)]\delta (\Phi )\left|\nabla \Phi \right|v_{n}d\Omega$$

Because the problem of structural stiffness maximization is a self-adjoint problem, we can create the following formula:

(22) $$\int_{\Omega }\varepsilon^{Τ}(\dot{u})E\varepsilon (u)d\Omega =\int_{\Omega }[fu+div(pun)-\varepsilon^{Τ}(u)E\varepsilon (u)]\delta (\Phi )\left|\nabla \Phi \right|v_{n}d\Omega$$

By substituting Equation (22) into Equation (16), to obtain the shape derivative of the objective function *J*(*Φ*) with respect to pseudo-time *t,* we use

(23) $$\frac{\partial J(\Phi )}{\partial t}=\int_{\Omega }G(\Phi )\delta (\Phi )\left|\nabla \Phi \right|v_{n}d\Omega$$

with

(24) $$G(\Phi )=fu+div(pun)-\frac{1}{2}\varepsilon^{Τ}(u)E\varepsilon (u)$$

The shape derivative of the volume constraint with respect to pseudo-time *t* can also be obtained:

(25) $$\frac{\partial g(\Phi )}{\partial t}=\int_{\Omega }\delta (\Phi )\left|\nabla \Phi \right|v_{n}d\Omega$$

The topological shape evolution represented by the LSF is achieved by solving the Hamilton–Jacobi partial differential equation. The key solution is found via a suitable normal velocity *v_n_*. This paper takes the global weight coefficients as design variables. By substituting the HLSF into the Hamilton–Jacobi partial differential equation, the original space- and time-coupled Hamilton–Jacobi partial differential equation is decoupled into a system of ordinary differential equations. Additionally, the ordinary differential equation only for pseudo-time *t* is given as:

(26) $$(\varphi_{1}(x)-\varphi_{2}(x))\frac{\partial w(t)}{\partial t}+\left|\nabla \Phi \right|v_{n}=0$$

The normal velocity *v_n_* is obtained to drive the movement of *∂*Ω in the cell:

(27) $$v_{n}=-\frac{1}{\left|\nabla \Phi \right|}\frac{\left(\varphi_{1}(x)-\varphi_{2}(x))\partial w(t\right)}{\partial t}$$

By substituting Equation (27) into Equation (23):

(28) $$\frac{\partial J\left(\Phi \right)}{\partial t}=-\sum_{k=1}^{m}\int_{\Omega }G\left(\Phi \right)\left(\varphi_{1}\left(x\right)-\varphi_{2}\left(x\right)\right)\frac{\partial w_{k}\left(t\right)}{\partial t}\delta \left(\Phi \right)d\Omega$$

By applying the chain rule for *J*(*Φ*):

(29) $$\frac{\partial J(\Phi )}{\partial t}=\sum_{k=1}^{m}\frac{\partial J(\Phi )}{\partial w_{k}\left(t\right)}\frac{\partial w_{k}(t)}{\partial t}$$

Comparing Equation (28) with Equation (29), the sensitivity with respect to weight coefficients is formulated as:

(30) $$\frac{\partial J(\Phi )}{\partial w_{k}(t)}=-\int_{\Omega }G(\Phi )(\varphi_{1}(x)-\varphi_{2}(x))\delta (\Phi )d\Omega$$

In the same way, by substituting Equation (27) into Equation (25):

(31) $$\frac{\partial g\left(\Phi \right)}{\partial t}=\sum_{k=1}^{m}\int_{\Omega }\left(\varphi_{1}\left(x\right)-\varphi_{2}\left(x\right)\right)\frac{\partial w_{k}\left(t\right)}{\partial t}\delta \left(\Phi \right)d\Omega$$

By applying the chain rule for *g*(*Φ*):

(32) $$\frac{\partial g(\Phi )}{\partial t}=\sum_{k=1}^{m}\frac{\partial g(\Phi )}{\partial w_{k}(t)}\frac{\partial w_{k}(t)}{\partial t}$$

Comparing Equation (31) with Equation (32) the sensitivity with respect to weight coefficients is formulated as:

(33) $$\frac{\partial g(\Phi )}{\partial w_{k}(t)}=\int_{\Omega }\left(\varphi_{1}(x)-\varphi_{2}(x)\right)\delta (\Phi )d\Omega$$

## 4. Numerical Implementation

**Figure 6:**
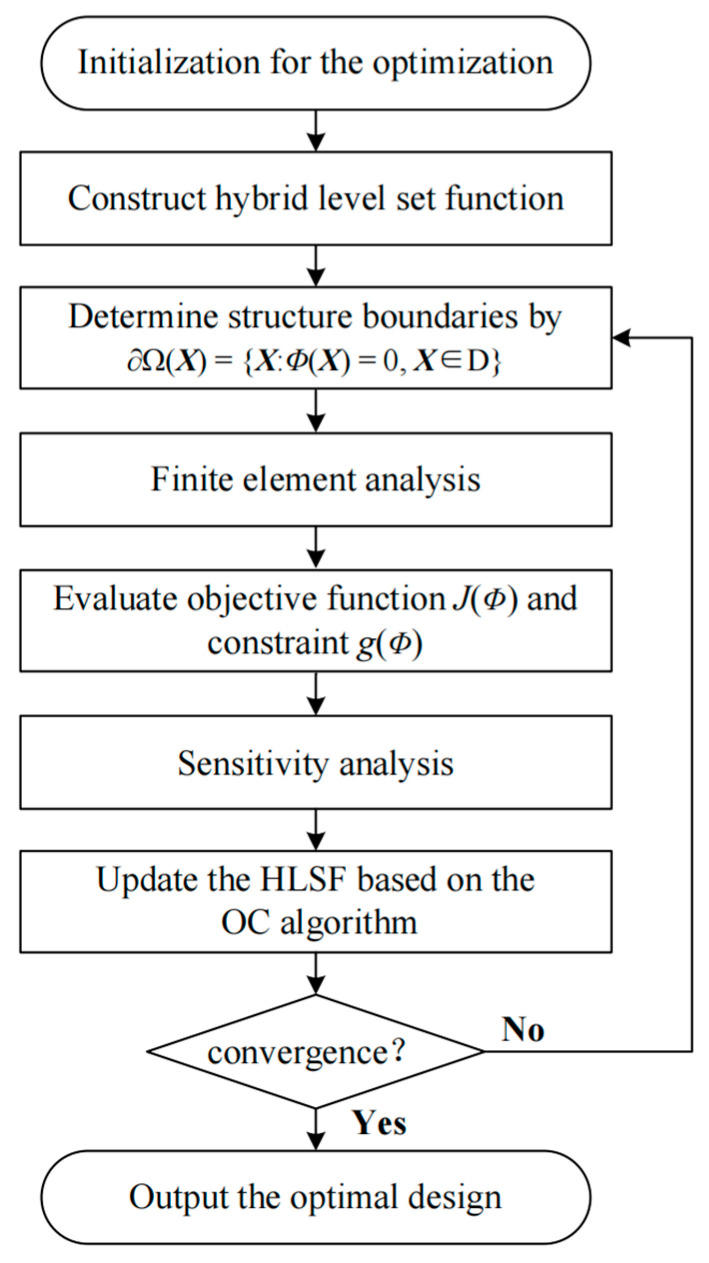
Flowchart of the optimization procedure.

The flowchart of the topology optimization of FGSs is shown in Figure 6. The optimization process begins with the initialization of the HLSF, including the structure mesh, definition of global weight coefficients, and pre-definition of unit cells, etc. Then, the initial unit cells are obtained based on Equation (11). During the optimization, the state equation in Equation (12) is solved to obtain the displacement field in each iteration. The sensitivity information is calculated based on the structural displacement field. The gradient-based mathematical optimizer can be used to update the design variables. The optimality criteria (OC) [43] is particularly efficient for solving large-scale structural optimization problems with a single constraint. The OC algorithm is thus used to update the design variables. The optimization terminates when the absolute difference of two successive objectives is less than 1 × 10^−3^ or the maximum loop number is reached.

## 5. Numerical Examples

In this paper, two typical numerical examples are presented to illustrate the effectiveness and applicability of HLSM for the topology optimization of FGSs, including TWSSs and FGCSs. In the optimization examples, the volume fraction of the structure is taken as the constraint condition, and the objective function is to maximize the structural stiffness.

### 5.1. Thin-Walled Stiffened Structures

In this example, HLSM is used to optimize the TWSSs. The optimal shape or topology configuration of the stiffening ribs depends on the given loading and boundary conditions. It has to be noted that, if there are no geometric constraints on the level set evolution, stiffening ribs with irregular shape and non-uniform wall thickness will be obtained. The free-form stiffening ribs create challenges in manufacturing, leading to an optimization result with no practical value. Taking into account the restrictions of manufacturing techniques, this paper introduces a geometry constraint to control the thickness of the stiffening rib in one specified spatial direction.

**Figure 7:**
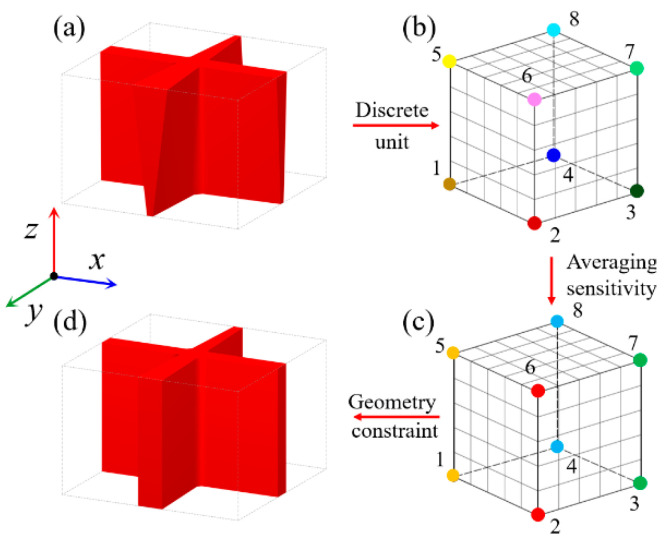
Geometry constraints on stiffening rib unit cell. (**a**) Unit cell without geometry constraint. (**b**) Discrete elements with different sensitivities. (**c**) Discrete elements with average sensitivities. (**d**) Unit cell with geometry constraint.

The geometry constrain is applied so that the optimized stiffening ribs have a uniform wall thickness in the direction that is perpendicular to the panel surface. For a unit cell discretized by eight-node hexahedral elements, the sensitivity values on each node are usually different in each sensitivity analysis process. A possibly optimized stiffening rib unit cell without any constraints is shown in Figure 7a. The global weight coefficients on the eight node of the unit cell are different in the Z-direction are plotted in Figure 7b. The geometric shape in the Z-direction is irregular, and the wall thickness is non-uniform. To control the shape or wall thickness in the Z-direction, the nodal sensitivities on the same line in the Z-direction are averaged. Thus, the design variables (global weight coefficients) on the same line in the Z-direction are of the same value. In this case, the stiffening rib unit cell shows a regular geometric shape or uniform thickness, as shown in Figure 7c,d. According to Equation (34), the average derivatives of the objective function with respect to the design variable are given as follows:

(34) $$\left\{\frac{\partial \bar{J}(\Phi )}{\partial w_{1}(t)}=\frac{\partial \bar{J}(\Phi )}{\partial w_{2}(t)}=\cdots =\frac{\partial \bar{J}(\Phi )}{\partial w_{NE}(t)}\right\}\;=\frac{1}{NE}{\sum_{i}^{NE}\frac{\partial J(\Phi )}{\partial w_{i}(t)}}_{j}\;j=1,2,\;\cdots ,\;\;N\;$$

where ∂*J*(*Φ*)/*w_i_*(*t*) is the original sensitivity of the objective function *J*(*Φ*) with respect to the design variable ***w***(*t*), which is on the node of the parallel element in the Z-direction. ∂*J*(*Φ*)/*w_i_*(*t*) is the averaged sensitivities. *NE* is the number of nodes (global design variables) in the Z-direction of unit cell. *N* is the number of unit cell nodes (global design variables) on the same layer section. The sensitivity constraints can perfectly control the geometry of the stiffener unit cell, as shown in Figure 7c,d. It should be noted that the sensitivity averaging operation is included in the optimization process of the TWSSs, rather than the post-processing. Therefore, it does not affect the mechanical properties and simulation accuracy of the optimized results. On the contrary, the proposed method ensures the regular geometry shape of stiffening ribs and effectively improves the manufacturability of the optimized results.

**Figure 8:**
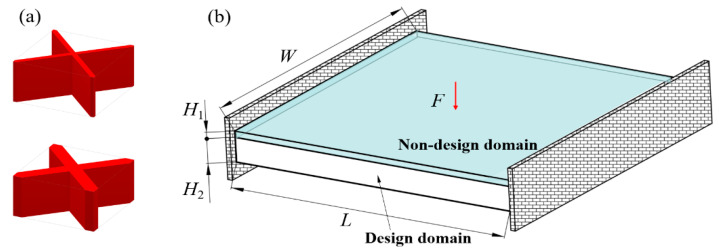
Pre-defined unit cells and design domain of TWSS. (**a**) X-shaped stiffening rib unit cells with different volume fractions. (**b**) Loading and boundary conditions for 3D TWSS.

The pre-defined X-shaped stiffening rib unit cells are shown in Figure 8a. Their volume fractions are 0.15 and 0.4, respectively. Figure 8b shows the design domain of the TWSS. The design domain is a square plate with *L* = 180, *W* = 180, *H*_1_ = 2, and *H*_2_ = 30. The upper thin wall is the non-design domain, and the lower thin wall is the design domain. The structure is fixed at both the left and right side. A concentrated load *F* = 10 is applied to the center of the upper surface of the structure. The design domain is discretized into 6 × 6 × 1 unit cells, and each unit cell is further discretized by 30 × 30 × 32 eight-node hexahedral elements. The upper thin wall is the non-design panel and contains two layers of elements. The volume fraction of the stiffened structure is set to *μ* = 0.3.

**Figure 9:**
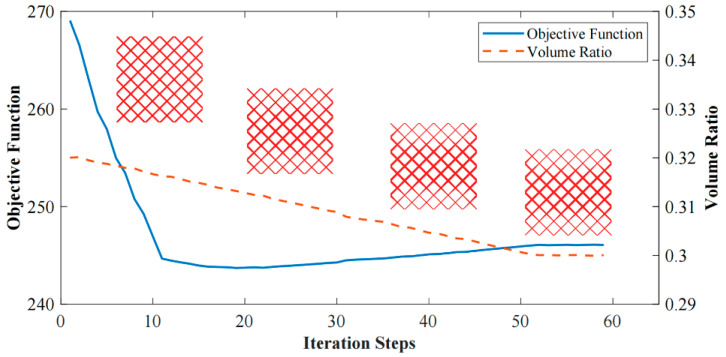
Interaction history of 3D TWSS design.

After 59 iteration steps, the optimization objective converges to *J* = 246; the iteration history is shown in Figure 9. As optimization continues, the material is gradually distributed on the force transfer paths. Less material is distributed in other positions. According to the optimization iteration history, the proposed optimization algorithm for TWSSs runs stably and converges well.

**Figure 10:**
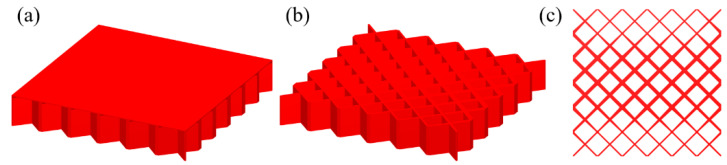
Optimized TWSS using the HLSM. (**a**) Optimized TWSS. (**b**) Optimized layout of stiffening ribs. (**c**) Top view of stiffening ribs.

The final optimized structure is presented in Figure 10a. The upper thin-wall structure is hidden to observe the detailed features of the stiffening ribs, as shown in Figure 10b,c. The optimized stiffened structure shows a clear gradient distribution in geometry and density, and no mismatch between graded cells is observed. In addition, the optimized stiffened structure has a uniform wall thickness by introducing shape constraints, so that the manufacturability is guaranteed.

To further verify the accuracy of the method proposed in this paper, the FEA is performed on the original and optimized TWSSs with the same volume fraction. The loading and boundary conditions are the same as those specified in Figure 8b. The deformation is used to evaluate the stiffness performance of stiffened structures. The design and FEA parameters of the geometry are listed in Table 1.

**Figure 11:**
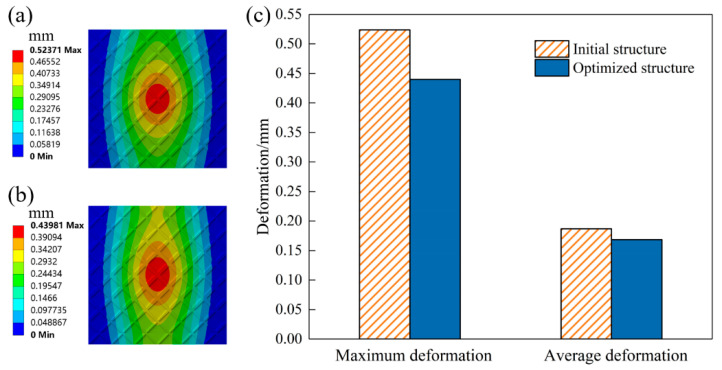
FEA results of TWSS. (**a**) Deformation nephogram of TWSS with uniform stiffening ribs layout. (**b**) Deformation nephogram of TWSS with optimized graded stiffening ribs layout. (**c**) Comparison of the FEA results.

The deformation nephograms of the two structures are shown in Figure 11a,b. The whole structural deformations are relatively similar. The fixed side deformation of the thin-walled stiffened structure is small. The large deformation is mainly concentrated in the center of the structures. The comparison of the two structural deformations is shown in Figure 11c. The maximum deformation (MD) decreases from 0.52371 mm to 0.43981 mm. The maximum deformation of the optimized TWSS reduces by 16.02%. The averaged deformations (AD) of the two structures are 0.18688 mm and 0.16853 mm, respectively. The averaged deformation is reduced by 9.82%. The simulation results of FEA further show that the proposed method can effectively realize the optimized layout of stiffening ribs and significantly improve the stiffness of TWSSs.

To further verify the applicability of the proposed method, cross-shaped and hybrid stiffening rib unit cells are pre-defined to design TWSSs, as shown in Table 2. The first and second columns of Table 2 are pre-defined stiffening rib unit cells and optimized topology configurations of TWSSs, respectively. The FEA is also performed on the uniform and optimized TWSSs with the same volume fraction. The deformation nephograms of the two structures are shown in the third and fourth columns of Table 2. Note that the proposed method is controlled by the geometry constraint and ensures the regular geometry shape of the stiffening ribs. The numerical examples in the Supplementary show that the proposed method can effectively realize an optimized layout of stiffening ribs with different forms and simultaneously enhance the mechanical properties of TWSSs.

### 5.2. Functionally Graded Cellular Structures

#### 5.2.1. Graded Cellular Structure

**Figure 12:**
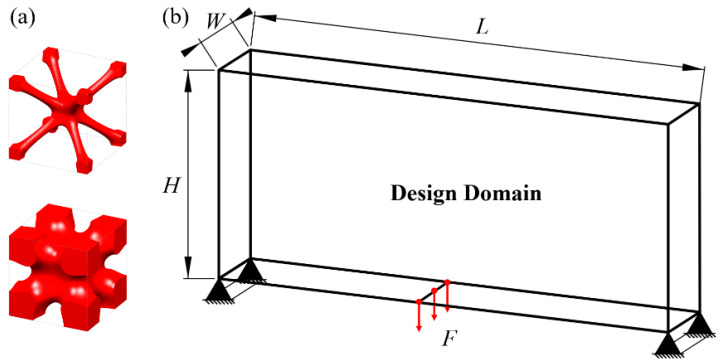
Pre-defined unit cells and design domain of FGCS. (**a**) I-WP TPMS with different volume fractions. (**b**) Loading and boundary conditions for 3D Michell beam.

In this example, HLSM is used to optimize the Michell beam structure to obtain FGCS. Considering that TPMSs have the advantages of a low density and high specific strength, as well as perfect self-supporting ability and chip removal ability in additive manufacturing (AM), the I-WP TPMS is used as the RVE of the cellular structure, as shown in Figure 12a, with volume fractions of 0.06 and 0.6, respectively. The loading and boundary conditions for the 3D Michell beam are illustrated in Figure 12b. The design domain is a cuboid with *L* = 240, *W* = 40 and *H* = 120. The distributed force *F* = 10 N is applied vertically downward in the middle of the bottom face. The bottom support edges located at the left and right face are fixed. The design domain is discretized into 12 × 2 × 6 unit cells, and each unit cell is further discretized by 20 × 20 × 20 eight-node hexahedral elements. The volume fraction constraint is set to *μ* = 0.4.

**Figure 13:**
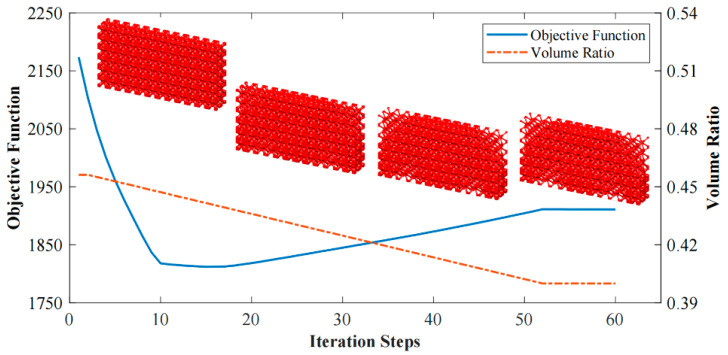
Interaction history of 3D Michell beam design.

**Figure 14:**
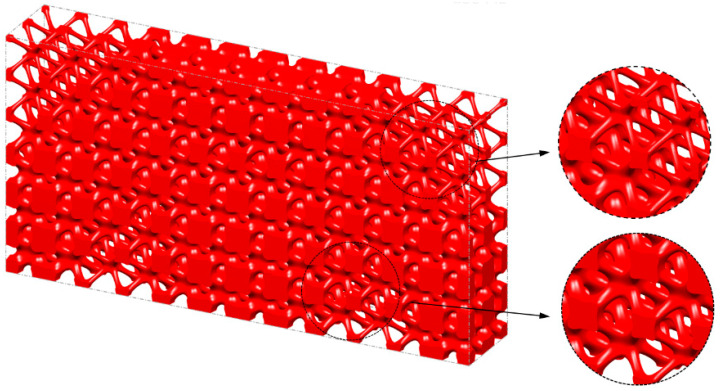
Optimized 3D Michell beam using the HLSM.

The optimization based on I-WP TPMS unit cells converges to *J* = 1910.96 after 60 iteration steps. The evolving history is plotted in Figure 13. Figure 14 shows the final design with a significant graded distribution in geometry and density. High-density unit cells with a high stiffness are distributed in the support domain, loading domain and force transfer paths to obtain superior mechanical properties. In addition, the perfect continuities between graded cells are guaranteed through the HLSM, where no mismatch is observed.

**Figure 15:**
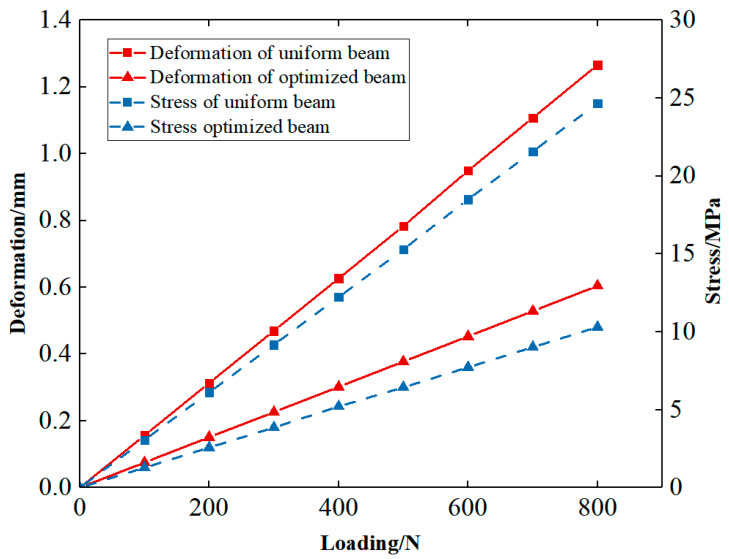
Comparison of maximum deformation and stress between uniform cellular structure and optimized FGCS.

**Figure 16:**
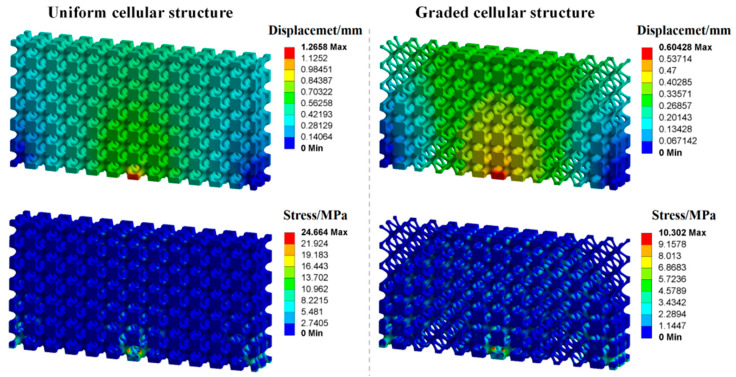
Deformation and stress nephograms of uniform cellular structure and optimized FGCS.

To illustrate the excellent mechanical properties of the optimized structure, the FEA is carried out for the uniform cellular structure and the FGCS under the same global volume fraction of 0.4. Loading and boundary conditions of structures for FEA are the same as Figure 12b. Nylon PA2200 is taken as the material for the geometry. The elastic modulus is 741 MPa, the yield strength is 54 MPa, the Poisson’s ratio is 0.3, and the density is 1020 kg/m^3^. The loading–deformation and loading–stress curves for the two structures are plotted in Figure 15. In the linear elastic region, the maximum deformation and stress of the uniform cellular structure are significantly larger than those of the optimized FGCSs. The stiffness of the structures is calculated according to the loading–deformation curve, which is 631.01 N/mm and 1323.94 N/mm, respectively. The FEA results show that the proposed method significantly improves the bearing capacity of cellular structure. Figure 16 shows the deformation and stress nephograms of the uniform cellular structure and the optimized FGCS under a loading condition of 800 N. Compared with the uniform cellular structure, more materials are distributed in the area of high stress for FGCS. This strategy will help us to improve the utilization ratio of the materials and load-bearing property of the macrostructure. It should be noted that the unit cells distributed in the cellular structure all evolve from the I-WP TPMS. The configurations of unit cells are similar in geometry, which may restrict the design space and its properties to some extent. However, they are still flexible enough for similar unit cells to achieve satisfactory properties and various functionalities [44,45].

When the minimum volume fraction of the pre-defined unit cell is greater than 0, a series of cells with different volume fractions are distributed throughout the design domain, as shown in the first row of Table 3. Therefore, it seems that the method proposed in this paper can only achieve the shape optimization of the structure. In fact, by simply adjusting the configuration and volume fraction of the pre-defined unit cells, the HLSM can achieve different optimization effects. For example, the topology optimization of cellular structure can be achieved when the minimum volume fraction of the pre-defined unit cell is 0. Furthermore, HLSM is also an effective tool to achieve macroscopic topology optimization using predefined unit cells with volume fractions of 0 and 1. The second and third rows of Table 3 show the topology evolution forms of the structures. It can be seen that the HLSM has significant advantages in the design of FGSs. Note that, small burrs appear on the boundaries of FGCS when using a unit cell with a volume fraction of 0. To avoid this problem, a pre-defined unit cell with a volume fraction greater than 0 is suggested, ensuring the mechanical properties and manufacturability of cellular structures.

#### 5.2.2. Lattice Sandwich Structure

**Figure 17:**
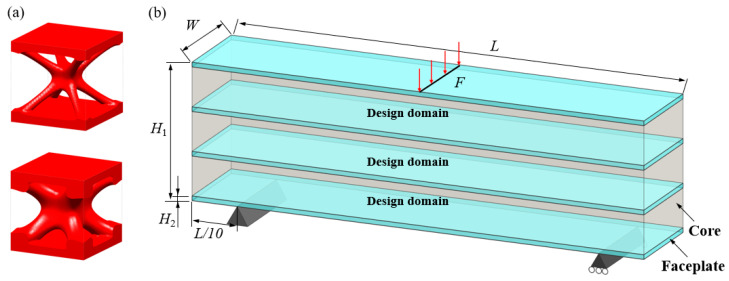
Pre-defined unit cells and design domain of LSS. (**a**) LSS unit cells with different volume fractions. (**b**) Loading and boundary conditions for 3D LSS.

Generally, the body-centered cube (BCC) lattice unit cell with rod-shaped parts is generally adopted to design the core layer of lattice sandwich structures (LSSs). However, existing studies show that the TPMS-based BCC has better load-bearing and energy-absorbing properties than those of the BCC with rod-shaped parts [46]. Therefore, I-WP TPMS is used as the core layer of LSSs in this section. Then, a novel graded lattice sandwich structure (GLSS) design is obtained based on the proposed HLSM. Figure 17a shows the pre-defined unit cells of LSS; their volume fractions are 0.25 and 0.45, respectively. The loading and boundary conditions of the LSS are illustrated in Figure 17b. Additionally, the sizes of the LSS are as follows: *L* = 240, *W* = 24, *H*_1_ = 96, *H*_2_ = 3. The core layer is the design domain and the thin and hard panels are non-design domains. The support domain is *L*/10 from both sides of the bottom of the design domain. The distributed force *F* = 10 N is applied vertically downward in the middle of the upper face. The design domain is discretized into 10 × 1 × 3 unit cells, and each unit cell is further discretized by 24 × 24 × 4 eight-node hexahedral elements. The volume fraction of the LSS is set to *μ* = 0.35.

**Figure 18:**
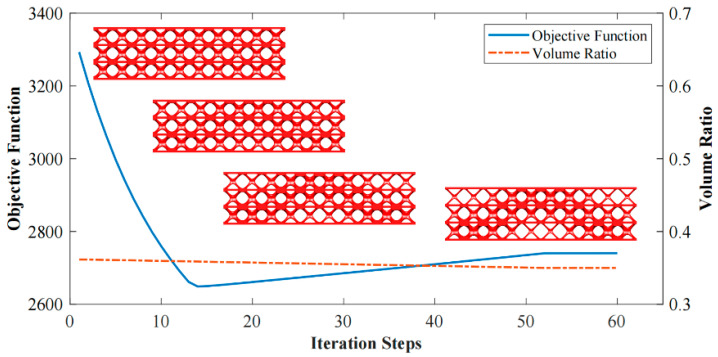
Interaction history of 3D LSS design.

**Figure 19:**
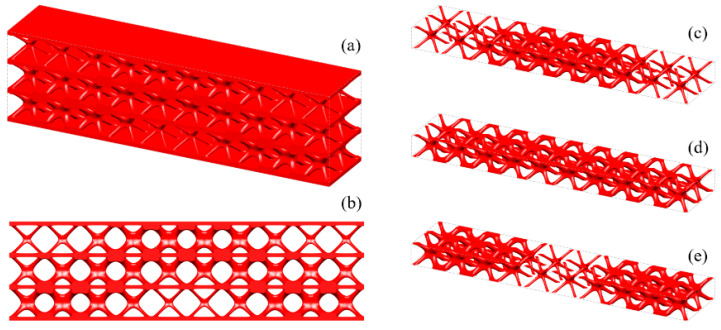
Optimized GLSS using the HLSM. (**a**) Optimized GLSS. (**b**) Front view of GLSS. (**c**) First core layer of GLSS. (**d**) Second core layer of GLSS. (**e**) Third core layer of GLSS.

Figure 18 shows the iterative curves of the objective function and volume fraction of the LSS. After 60 iteration steps, the objective function converges to *J* = 2704.38 under the global volume fraction constraint. The optimized GLSS is plotted in Figure 19. High-density unit cells with high stiffness pave the way from the loading domain to the support domain. Some low-density unit cells are distributed in the secondary force transfer paths to stably support the upper and lower solid faceplates. According to Figure 19c–e, the geometry configuration and volume fraction of each core layer are different. The core layers show graded changes according to the external loading and boundary conditions. Hence, the GLSS has a better bearing capacity.

## 6. AM and Experimental Validation

To further illustrate the effectiveness of the HLSM, samples of LSSs with different structural forms are fabricated by AM. Then, three-point bending tests and a simulation analysis are carried out to evaluate the mechanical performance of the three LSSs.

### 6.1. Geometric Modeling and AM Process

**Figure 20:**
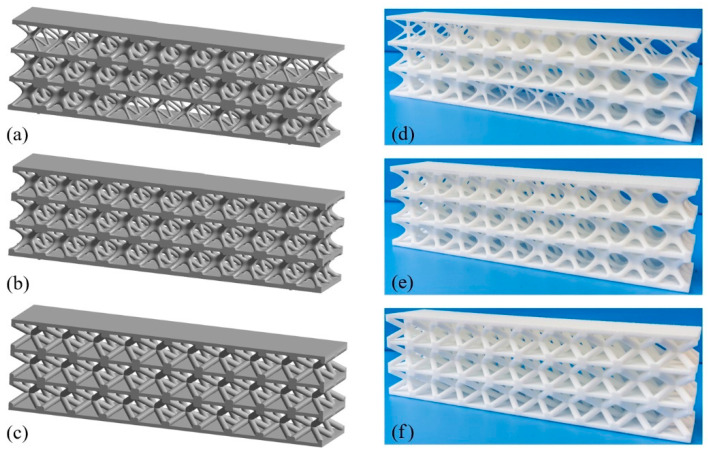
CAD models and printed structures of the test samples. (**a**) CAD model of optimized GLSS. (**b**) CAD model of LSS with uniform I-WP TPMS. (**c**) CAD model of LSS with uniform BCC unit cells. (**d**) Printed sample of (**a**). (**e**) Printed sample of (**b**). (**f**) Printed sample of (**c**).

Three LSSs geometrical models with diverse lattice core layers are constructed, as shown in Figure 20. Figure 20a is the optimized GLSS model with a volume fraction of 0.35. For comparison, Figure 20b shows the uniform lattice sandwich structure (ULSS) with BCC based on I-WP TPMS, and Figure 20c shows ULSS with BCC using rod-shaped parts. The volume fraction of the two ULSSs is the same as that of the GLSS. The three models are half the size of the model shown in Figure 17b. Selective laser sintering technology is utilized to manufacture the LSS samples since it has the characteristics of a high manufacture efficiency and high processing quality. The machine model is an EOS-P760 3D printer, and the material used in the AM process is PA2200. The machine uses a CO2 laser with the laser power of 50 W, the scanning speed of 6000 mm/s, layer thickness of 0.1 mm, and 100% filling rate. The test samples are shown in Figure 20d–f.

### 6.2. Mechanical Test

**Figure 21:**
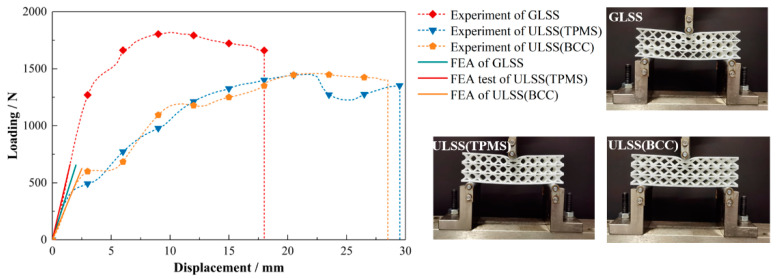
Loading and displacement curves from three-point bending tests of the printed LSSs.

**Figure 22:**
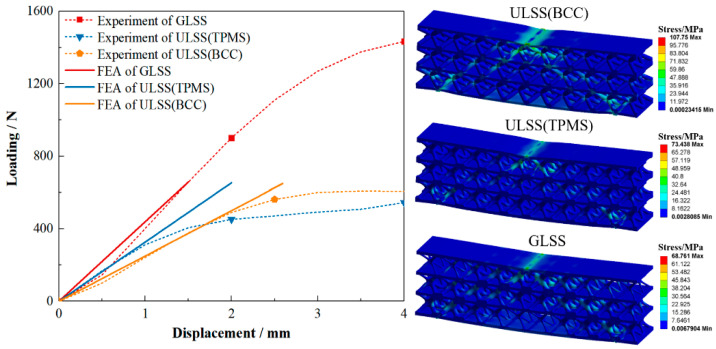
Comparison between the FEA and experimental results for the LSSs.

All the experiments are performed on an electronic universal testing machine. The loading rate is set as 0.5 mm/min until failure to simulate the quasi-static compression condition. A data acquisition system of the testing machine is utilized to record the displacements and corresponding loads during the loading process. The experimental load–displacement curves, as well as the boundary and load conditions of the test samples, are shown in Figure 21. Note that the load–displacement slope of the optimized GLSS is steeper than those of the two ULSSs under the same volume fraction. It indicates that the optimized structure has a higher stiffness, and the proposed method works well for compliance minimization problems. Furthermore, the strength of GLSS also performs better than that of ULSSs since the peak load of the GLSS is much larger than that of the ULSS. FEA is also carried out to evaluate the GLSS and ULSSs, as shown in Figure 22. It can be seen that the stiffness of optimized GLSS obtained by the FEA is close to the experimental results. For the two ULSSs, the load–displacement slope of the ULSS with I-WP TPMS is steeper before the buckling, indicating that the initial stiffness is higher than that of the ULSSs using rod-shaped parts. Overall, the proposed HLSM for LSSs can generate graded core layers and achieve their reasonable arrangement to explore the design potential of LSSs with utmost accuracy.

## 7. Conclusions

This paper presents a hybrid level set method for the topology optimization of FGSs, including TWSSs and FGCSs. The affine concept of convex optimization theory is introduced to construct HLSF. Controlled by the weight coefficients defined on element nodes, the HLSF can be optimized to generate the graded pattern in the macro scale for the FGSs. By presetting the level set functions of different structural unit cells, various FGSs are obtained with excellent graded patterns and significantly decreased structure compliance. In general, the proposed method has the following advantages:

**(1) High versatility and effectiveness:** This paper constructs a design strategy to drive the topology optimization of macro/microstructures. By presetting the level set functions of various structural unit cells, high-performance FGSs with different structural forms and functional properties could be obtained using level set evolution. Hence, HLSM is a convenient tool for designers.

**(2) Perfect geometric continuity:** According to the definition of weight coefficients, neighboring unit cells share the same global weight coefficients on common boundaries. Additionally, the local design variables are updated by the interpolation of global design variables. Thus, the interpolation function of the global weight coefficients has at least C^0^ continuity. With this updating strategy of design variables, the proposed HLSM can naturally guarantee perfect geometry continuity without imposing any extra constraints.

**(3) Easy-to-manufacture:** For TWSSs, the geometry constraint is proposed to ensure regular geometry shape of the stiffening ribs, so that TWSSs can be manufactured by welding or casting processes. Hence, excellent manufacturability is obtained. For FGCSs, the self-supporting lattice unit cell could be employed to design FGCSs, which are easy to fabricate through AM.

TWSSs and FGCSs are investigated to demonstrate the effectiveness and applicability of the proposed method. The numerical and experimental results show that the obtained FGSs have better mechanical performance compared with the uniform structure. Therefore, the proposed HLSM has great potential and competitiveness for the design of FGSs. Although only the static bearing capacity of the FGSs is studied in this paper, the proposed HLSM is expected to be effective in the generative design of functional structures in multi-physics. Fu, J.; Shu, Z.; Gao, L.; Zhou, X.

## Figures and Tables

**Table 1:** Design and FEA parameters of the geometry.

Model Size				Material Properties		Loading
*W*	*L*	*H*_1_	*H*_2_	Elastic Modulus	Poisson Ratio	Concentrated Load
180 mm	180 mm	2 mm	5 mm	2 × 10^5^ MPa	0.3	1000 N

**Table 2:** Micro-structural and macro-structural topologies and their FEA results.

Pre-Defined Unit Cells	Topological Configuration	Uniform Structure	Optimized TWSS
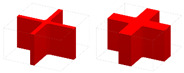	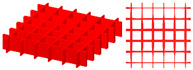	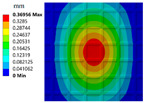	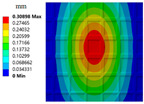
Volume fractions: 0.15, 0.4	*μ* = 0.3, *J* = 229	MD: 0.37 mm AD: 0.12 mm	MD: 0.31 mm AD: 0.09 mm
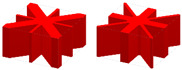	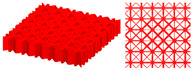	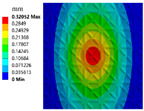	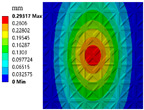
Volume fractions: 0.4, 0.5	*μ* = 0.45, *J* = 215	MD: 0.32 mm AD: 0.11 mm	MD: 0.29 mm AD: 0.09 mm

**Table 3:** The influence of pre-defined unit cells with different volume fractions and topological configurations on optimized results.

Pre-Defined Unit Cells	3D Michell Beams	3D Cantilever Beams